# Marked response to dabrafenib plus trametinib in a patient with BRAF V600E-mutant pancreatic hepatoid carcinoma: a case report and systematic analysis of 57 cases

**DOI:** 10.3389/fonc.2026.1927414

**Published:** 2026-08-18

**Authors:** Yingli Li, Shengzhi Xie, Xianchang Sun, Fangwei Wang, Hanbing Guo, Di Chen, Jianhua Dang, Xiaoli Yang, Yufei Wang, Xueping Ma

**Affiliations:** The Third Medical Center of Chinese PLA General Hospital, Beijing, China

**Keywords:** alpha-fetoprotein, BRAF V600E, dabrafenib, pancreatic hepatoid carcinoma, systematic analysis, targeted therapy, trametinib

## Abstract

**Background:**

Pancreatic hepatoid carcinoma (PHC) is an extremely rare pancreatic malignancy characterized pathologically by hepatocellular-like differentiation. Some patients may present with elevated serum alpha-fetoprotein (AFP). Owing to the limited number of reported cases, the clinical features, molecular characteristics, and systemic treatment strategies for PHC remain poorly defined. BRAF V600E is an actionable alteration with established therapeutic value in several solid tumors; however, its clinical significance in PHC remains unclear.

**Case presentation:**

We report the case of a 64-year-old man with advanced PHC who presented with painless jaundice, dark urine, and recent weight loss. Laboratory tests showed marked cholestatic liver injury and significantly elevated AFP. Imaging revealed a pancreatic head-neck mass with portal vein tumor thrombus and regional lymph node metastases, corresponding to cT4N1M1, stage IV disease. Percutaneous transhepatic biliary drainage was first performed to relieve obstructive jaundice. Biopsy of the pancreatic lesion showed poorly differentiated carcinoma. Based on hepatoid morphology, immunophenotype, elevated serum AFP, imaging findings, and exclusion of primary hepatocellular carcinoma, the patient was diagnosed with PHC. Comprehensive genomic profiling identified a BRAF V600E mutation with a variant allele frequency of 31.89%, together with MDM2 and MYC amplification. The molecular profile was characterized by microsatellite stability, low tumor mutational burden, MGMT promoter methylation, and low PD-L1 expression. After two cycles of pembrolizumab-based first-line therapy combined with paclitaxel, S-1, and lenvatinib, AFP continued to increase and imaging showed rapid tumor enlargement, consistent with immune checkpoint inhibitor-related hyperprogressive disease. The treatment was then switched to dabrafenib plus trametinib. AFP declined rapidly and returned to the normal range within approximately two months. Imaging showed marked regression of the pancreatic primary lesion, disappearance of the portal vein tumor thrombus and metastatic lymph nodes, and conversion of peripheral blood minimal residual disease to negative. The best response was partial response. After approximately six months of targeted therapy, occult disease progression emerged. Subsequent addition of cetuximab, replacement of the MEK inhibitor, and dose escalation of targeted therapy did not restore sustained systemic disease control, although local disease remained manageable with subsequent treatment adjustments. Proton radiotherapy was then delivered to the residual pancreatic lesion, followed by CyberKnife radiotherapy for a newly detected 2.3-cm metastasis in the caudate lobe of the liver. As of April 2026, the patient’s AFP level remained close to normal at 14 ng/mL, local lesions were well controlled, peripheral blood minimal residual disease had turned positive, and the patient remained in a stable tumor-bearing state.

**Systematic analysis:**

We further summarized 57 previously reported cases of PHC. The median age was 54 years, and 66.7% of patients were male. Tumors occurred at different pancreatic sites, including the pancreatic head in 21 cases, body in 8 cases, tail in 13 cases, and multifocal lesions in 15 cases. More than half of the patients had metastatic disease at initial diagnosis. The immunophenotype of PHC was highly heterogeneous. Regarding treatment, 47 patients underwent surgery, 20 received chemotherapy, and 6 received targeted therapy. The 1-year and 3-year overall survival rates were 70.7% and 43.1%, respectively, indicating an overall poor prognosis.

**Conclusion:**

This case suggests that BRAF V600E may represent a clinically actionable driver alteration in PHC. Dabrafenib plus trametinib induced a rapid and deep response in this patient with advanced BRAF V600E-mutant PHC. Microsatellite stability, low tumor mutational burden, low PD-L1 expression, and MDM2 amplification may be associated with limited benefit from immunotherapy and a risk of hyperprogression. After resistance to targeted therapy, local radiotherapy may serve as an important strategy for controlling oligoresidual and oligometastatic lesions. Together with the literature review, this case supports early comprehensive molecular profiling and individualized multidisciplinary management for advanced PHC.

## Introduction

Hepatoid carcinoma is a rare malignant neoplasm characterized by hepatocellular differentiation occurring outside the liver. It has been reported in various organs, including the stomach, lung, ovary, pancreas, and other extrahepatic sites. These tumors are frequently associated with elevated serum alpha-fetoprotein (AFP) levels and are generally characterized by aggressive biological behavior and a high propensity for metastasis. However, AFP elevation is not required for diagnosis. A definitive diagnosis relies on the integration of histopathological findings, immunophenotypic features, serological markers, and radiological evidence (1).

Pancreatic hepatoid carcinoma (PHC) is an exceptionally rare subtype of pancreatic malignancy, with the current literature consisting primarily of isolated case reports and small case series. Owing to considerable overlap in morphological and immunohistochemical features with primary hepatocellular carcinoma, pancreatic ductal adenocarcinoma, acinar cell carcinoma, and neuroendocrine neoplasms, the diagnosis of PHC remains particularly challenging (2). Furthermore, previous studies have suggested that many patients present with locally advanced disease, lymph node involvement, or distant metastases at the time of diagnosis, resulting in an unfavorable prognosis. Surgical resection may provide meaningful long-term survival benefits in patients with resectable disease; however, no established standard systemic treatment currently exists for patients with locally advanced, metastatic, or recurrent PHC (3).

Currently, molecular classification and precision medicine are reshaping the treatment landscape of solid tumors. BRAF V600E is a classic driver mutation in the MAPK signaling pathway and has been established as an actionable target in several solid tumors. Combined BRAF and MEK inhibition exerts antitumor activity through dual blockade of the MAPK pathway, and multiple studies have shown its potential as a tumor-agnostic therapeutic strategy (4–6). However, direct clinical evidence regarding the prevalence of BRAF V600E mutations in PHC, their therapeutic relevance, and treatment strategies after acquired resistance remains limited. Meanwhile, advanced PHC may harbor molecular features such as microsatellite stability, low tumor mutational burden, low PD-L1 expression, and MDM2 or MYC amplification, which may influence the efficacy of immunotherapy and patterns of disease progression; however, current understanding remains insufficient.

Here, we report a case of advanced PHC harboring a BRAF V600E mutation. The patient experienced rapid progression after chemotherapy and subsequently achieved a deep response to dabrafenib plus trametinib. After evidence suggestive of occult progression emerged during the later phase of targeted therapy, subsequent systemic treatment adjustments provided limited additional disease control, whereas local radiotherapy played an important role in controlling both the residual pancreatic lesion and the subsequent oligometastatic liver lesion. At the last follow-up, the patient remained in a long-term stable tumor-bearing state. In addition, we systematically summarized 57 previously reported PHC cases and analyzed their clinicopathological features, treatment patterns, and survival outcomes, aiming to provide reference evidence for diagnostic recognition, molecular stratification, and individualized multidisciplinary management of PHC.

## Case presentation

A 64-year-old man presented in mid-February 2024 with painless yellowing of the skin and sclera, accompanied by tea-colored urine. He had experienced an approximately 10 kg weight loss over the preceding 2 months. His medical history included long-term smoking, hypertension, and previous resection of a right adrenal cortical adenoma. No definite family history of malignancy or hereditary disease was reported. Laboratory tests on admission showed marked cholestatic liver injury with multiple abnormal liver function parameters. Serum alpha-fetoprotein (AFP) was markedly elevated to 12,457 ng/mL, CA19–9 was mildly elevated, and other gastrointestinal tumor markers showed no obvious abnormalities.

**Figure 1:**
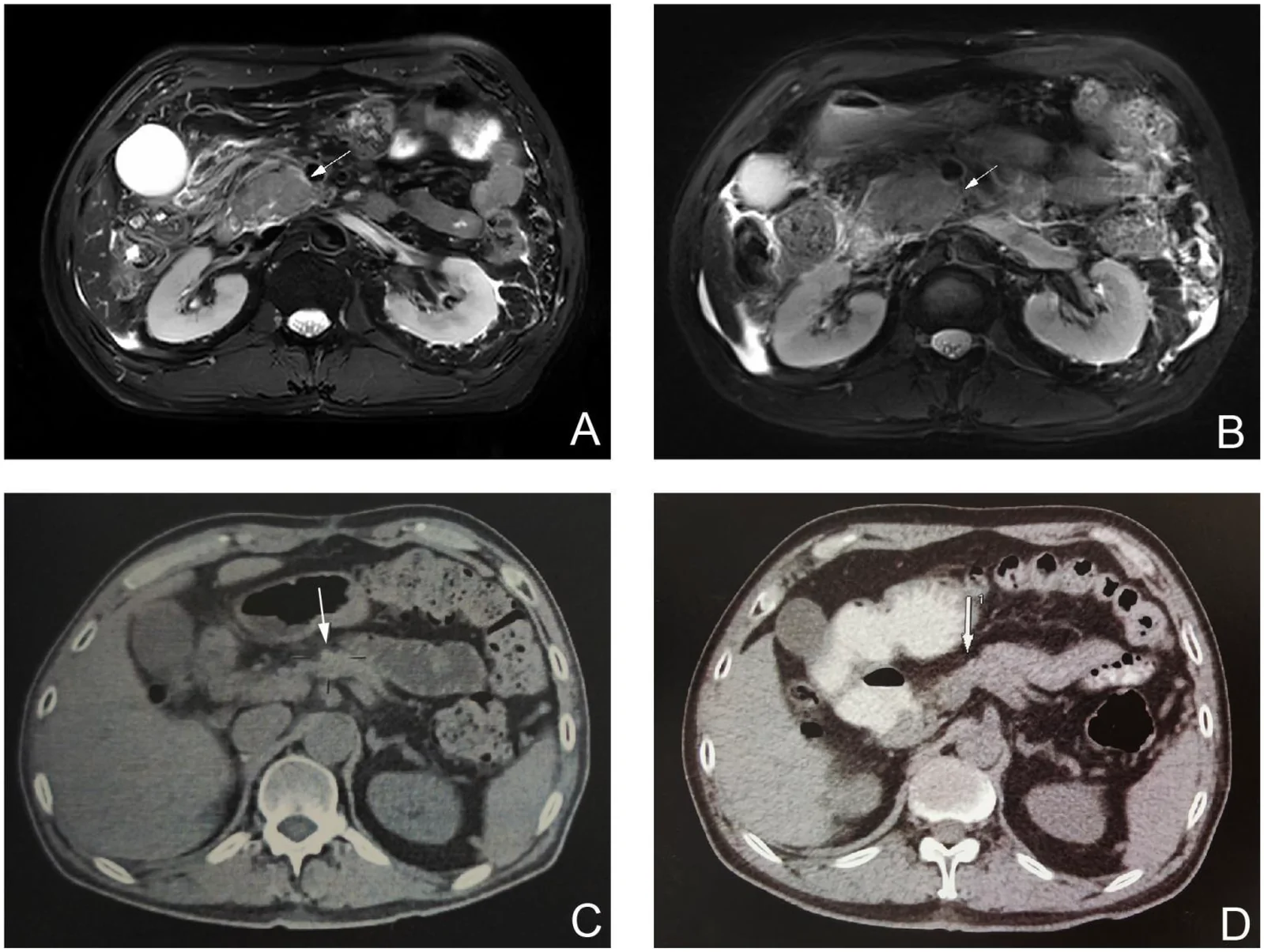
Serial abdominal imaging during treatment. **(A)** Baseline MRI obtained on February 21, 2024, showed a visible lesion involving the pancreatic head–neck region. **(B)** MRI obtained on March 21, 2024, after exploratory chemotherapy combined with immunotherapy showed no evident reduction in tumor size. **(C)** PET/CT obtained on July 27, 2024, showed marked regression of the pancreatic mass to approximately 0.7 cm with an SUVmax of 2.3, accompanied by substantial regression of portal vein tumor thrombosis and metastatic lymph nodes. **(D)** FAPI PET/CT obtained in October 2024 showed a residual lesion in the pancreatic neck region. White arrows indicate the target lesion.

Imaging in February 2024 revealed a mass measuring approximately 5.8 cm × 2.4 cm × 2.3 cm in the pancreatic head-neck region, accompanied by portal vein tumor thrombus and multiple abdominal lymph node metastases. No definite primary hepatic lesion was identified (**Figure 1A**). Based on the imaging findings, the clinical stage was considered cT4N1M1, stage IV. To relieve biliary obstruction, the patient underwent percutaneous transhepatic biliary drainage, after which bilirubin levels and liver function gradually improved.

**Figure 2:**
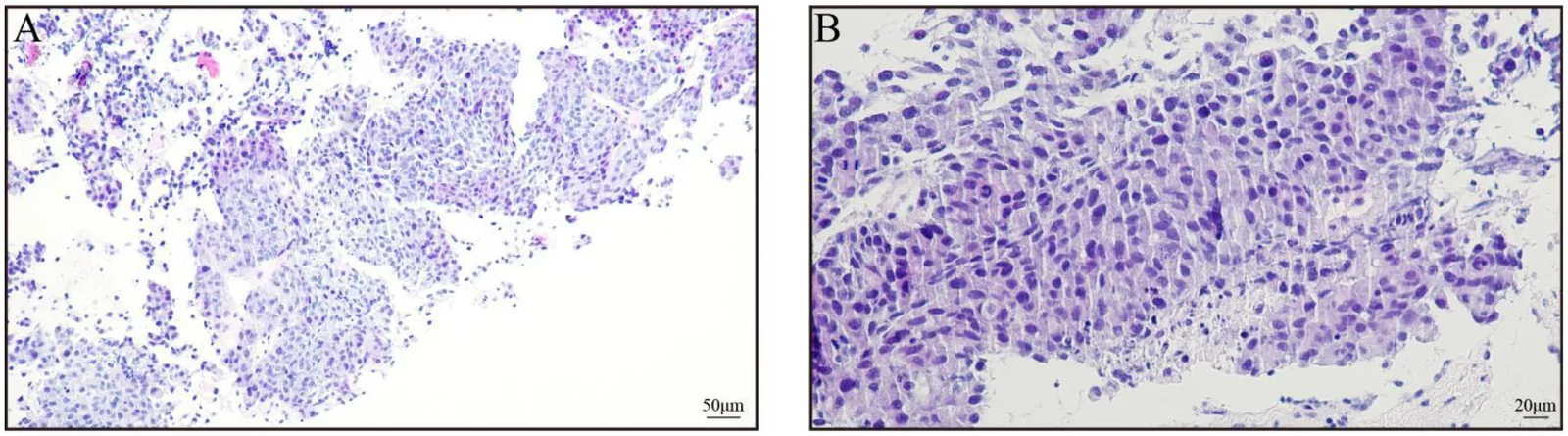
Pathological presentation of the patient. **(A)** Hematoxylin and eosin staining revealed heteromorphic neoplastic cells arranged in glandular, nested, and trabecular patterns at low magnification (magnification, ×100; scale bar, 50 μm). **(B)** High-power view showed atypical tumor cells with enlarged hyperchromatic nuclei and irregular glandular/nested growth patterns (magnification, ×200; scale bar, 20 μm).

Subsequently, a biopsy of the pancreatic lesion was performed. Pathological examination showed infiltration of poorly differentiated malignant tumor cells within fibrous tissue. The tumor cells had eosinophilic cytoplasm, marked nuclear atypia, and were arranged in nested and sheet-like patterns, with hepatoid morphology in some areas. Immunohistochemistry (**Figure 2**) showed that the tumor cells were positive for AFP, SALL4, CK, CK19, and synaptophysin, and negative for CK7, CD56, CEA, and INSM1, among other markers. The Ki-67 proliferation index was approximately 75%. The mismatch repair proteins MLH1, MSH2, MSH6, and PMS2 showed intact expression, indicating pMMR/MSS status; PD-L1 expression was low. Based on the markedly elevated serum AFP, absence of an intrahepatic primary lesion on imaging, pathological morphology, and immunophenotype, pancreatic metastasis from primary hepatocellular carcinoma, typical pancreatic ductal adenocarcinoma, and pancreatic neuroendocrine tumor were considered unsupported. The final diagnosis was pancreatic hepatoid carcinoma with focal expression of neuroendocrine markers. Further genomic testing identified a BRAF V600E mutation, accompanied by MDM2 and MYC amplification. The tumor had a low tumor mutational burden and positive MGMT promoter methylation.

From February 28 to April 12, 2024, the patient received pembrolizumab, lenvatinib, paclitaxel, and S-1, with each cycle lasting 21 days, for a total of two cycles. During treatment, he developed grade 2–3 myelosuppression and grade 1 gastrointestinal reactions, accompanied by decreased cortisol levels, oral ulcers, rash, and recurrent epistaxis. These adverse events improved after symptomatic management, and no obvious hepatic or renal impairment was observed. After two cycles, serum AFP increased to 18,000 ng/mL. Imaging showed enlargement of the pancreatic primary lesion and metastatic lymph nodes compared with baseline, with some lymph nodes increasing by approximately 40% (**Figure 1B**). Given the rapid increase in tumor burden and marked elevation of tumor markers within a short period, immune checkpoint inhibitor-related hyperprogressive disease was clinically considered. Therefore, the first-line regimen was promptly discontinued.

Given the presence of a BRAF V600E mutation, the patient was switched to oral targeted therapy with dabrafenib plus trametinib on April 13, 2024. After 1 week of treatment, AFP decreased to 9,400 ng/mL; after approximately 2 months, AFP returned to the normal range. PET/CT performed on July 27, 2024, showed marked shrinkage of the pancreatic mass to approximately 0.7 cm, with an SUVmax of 2.3. The portal vein tumor thrombus and metastatic lymph nodes had markedly regressed or disappeared (**Figure 1C**). The treatment response was assessed as partial response. In August 2024, plasma ctDNA-based MRD testing showed no detectable tumor-associated molecular signal, corresponding to an MRD-negative result (0 hGE/mL). Subsequent serial monitoring showed re-emergence of the molecular residual signal, with ctDNA levels increasing to 2.75 hGE/mL in November 2024 and 11 hGE/mL in April 2025, indicating that the molecular response was not sustained. The targeted regimen was generally well tolerated, with only mild fatigue and diarrhea that did not require specific drug intervention.

During continued dual BRAF/MEK-targeted therapy, AFP gradually increased again, suggesting possible occult residual disease or early progression. In October 2024, FAPI PET/CT showed a residual metabolically active lesion in the pancreatic neck (**Figure 1D**). Considering that systemic disease had been well controlled but a suspicious local residual lesion remained, the patient received proton radiotherapy from October to December 2024, delivered in 26 fractions with a prescribed target dose of 65 Gy. During radiotherapy, AFP decreased again to the normal range, and no intolerable adverse events occurred.

Thereafter, the patient continued to undergo subsequent treatment adjustments centered on the MAPK pathway. Owing to the persistent upward trend in AFP, dabrafenib dose escalation, cetuximab addition on the basis of BRAF/MEK inhibition, and replacement of trametinib with tunlametinib were performed sequentially. These individualized adjustments were made after multidisciplinary evaluation, taking into account the patient’s previous marked response to BRAF/MEK inhibition, treatment tolerance, the continued increase in AFP, and the limited availability of established salvage regimens for progressive PHC. Despite these adjustments, tumor markers were not durably controlled, raising the possibility of emerging resistance to targeted therapy, although no specific resistance mechanism was molecularly confirmed. Suggesting the development of resistance to targeted therapy. The related systemic therapy was ultimately discontinued in December 2025.

**Figure 3:**
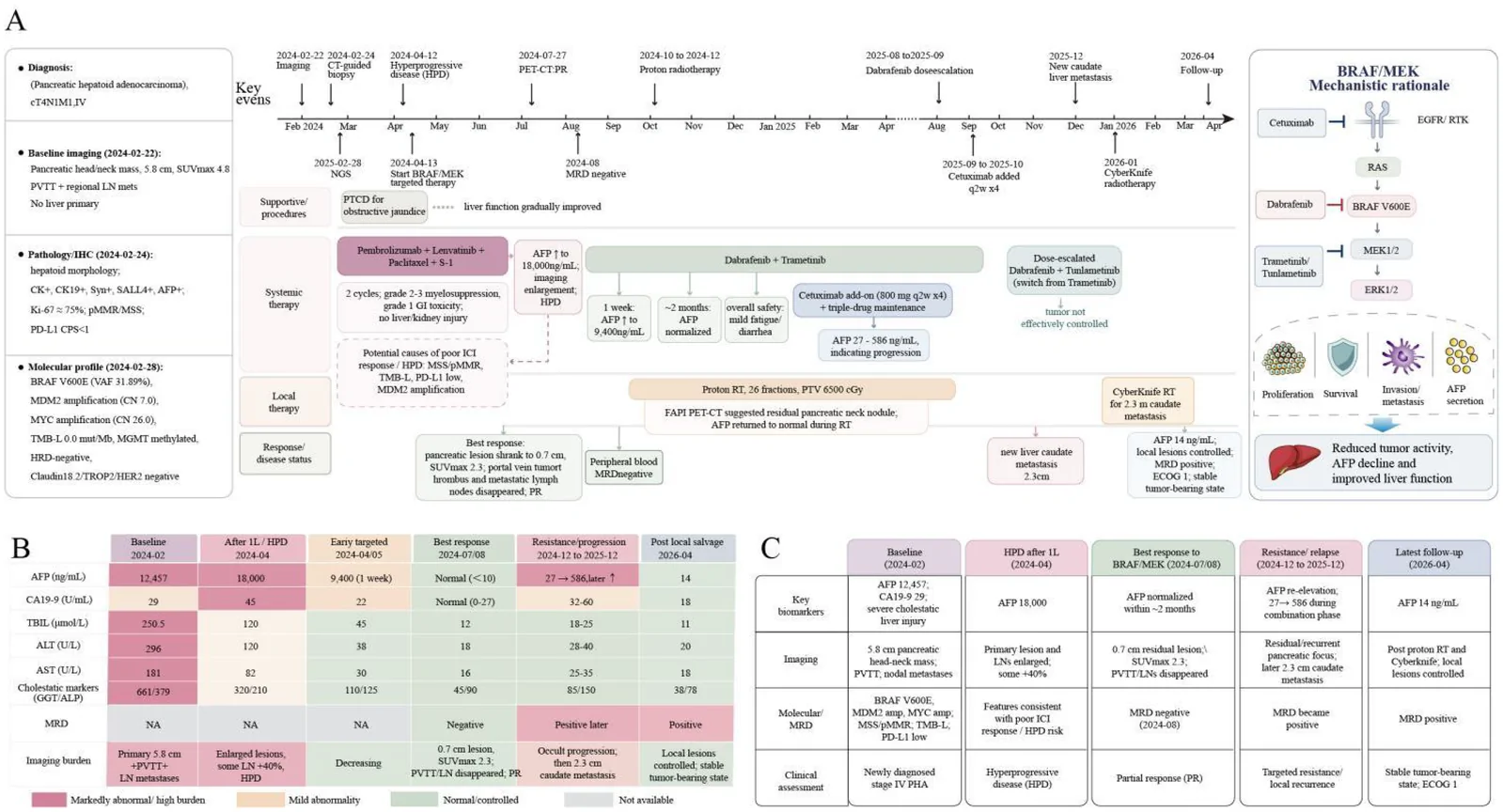
Therapeutic course, biomarker dynamics, and treatment rationale in a patient with pancreatic hepatoid adenocarcinoma. **(A)** Integrated timeline of diagnosis, molecular profiling, systemic therapy, local treatment, and disease response. The patient presented with stage IV pancreatic hepatoid adenocarcinoma harboring a BRAF V600E mutation. After hyperprogression following initial chemoimmunotherapy, BRAF/MEK-targeted therapy induced marked biochemical and radiological responses, followed by proton radiotherapy, cetuximab add-on treatment, BRAF/MEK dose adjustment, and CyberKnife radiotherapy for liver metastasis. The schematic illustrates the rationale for combined EGFR, BRAF, and MEK inhibition. **(B)** Longitudinal changes in serum biomarkers, MRD status, and imaging burden during treatment. AFP and cholestatic markers increased during hyperprogression but decreased substantially after BRAF/MEK-targeted therapy, accompanied by MRD negativity and radiological partial response. Subsequent AFP re-elevation and MRD positivity indicated resistance/progression. **(C)** Stage-by-stage summary of biomarkers, imaging findings, molecular/MRD status, and clinical assessment. The patient achieved partial response after BRAF/MEK-targeted therapy, developed later resistance with liver metastasis, and remained in a stable tumor-bearing state after local salvage treatment. AFP, alpha-fetoprotein; EGFR, epidermal growth factor receptor; HPD, hyperprogressive disease; MRD, minimal residual disease; PHA, pancreatic hepatoid adenocarcinoma; PR, partial response.

In December 2025, follow-up imaging revealed a newly developed metastatic lesion of approximately 2.3 cm in the caudate lobe of the liver, indicating further disease progression. Accordingly, the radiographic progression-free survival from the initiation of dabrafenib plus trametinib on April 13, 2024, to the first radiologically documented progression in December 2025 was approximately 20 months. Although serum AFP had begun to increase earlier, this biochemical change was not used alone to define radiographic progression. Given the limited extent of the new lesion, the patient underwent CyberKnife stereotactic radiotherapy in January 2026. At the last follow-up in April 2026, both the residual pancreatic lesion and the caudate lobe liver metastasis remained locally controlled. AFP had decreased to 14 ng/mL, and imaging showed no new metastatic lesions. Serial plasma ctDNA-based MRD monitoring subsequently became positive again, with an increase in the detected molecular residual signal. This finding suggested possible persistent or recurrent molecular disease. The patient currently remains in a stable tumor-bearing state, and subsequent treatment is still under further evaluation. The overall survival from the initial diagnosis in February 2024 to the last follow-up in April 2026 was approximately 26 months. Because the patient remained alive at the data cutoff, the OS observation was censored at the last follow-up. The complete diagnostic and treatment timeline, including initial diagnosis, first-line therapy, BRAF/MEK-targeted therapy, local radiotherapy, and last follow-up, together with changes in AFP, minimal residual disease, imaging response, stage-specific clinical assessment, and the mechanistic rationale for BRAF/MEK-targeted therapy, is summarized in **Figure 3**.

## Systematic analysis methods

### Literature search and selection

To systematically evaluate the clinicopathological characteristics, treatment patterns, and prognostic outcomes of pancreatic hepatoid carcinoma, we searched and screened previously published literature. The databases searched included PubMed and Web of Science. The search terms were “Pancreatic Hepatoid Carcinoma” or “Hepatoid Adenocarcinoma of the Pancreas”. To minimize omissions, we also manually reviewed previous review articles and the reference lists of eligible studies to identify additional relevant case reports.

The inclusion criteria were as follows: pathologically confirmed pancreatic hepatoid carcinoma; and availability of extractable individual patient-level data, including at least one of the following: clinical characteristics, pathological or immunohistochemical findings, treatment modalities, follow-up information, or survival outcomes. The exclusion criteria were duplicate cases, hepatoid carcinoma originating from non-pancreatic sites, lack of pathological confirmation, unavailable individual-level data, or insufficient full-text information to support systematic analysis.

**Figure 4:**
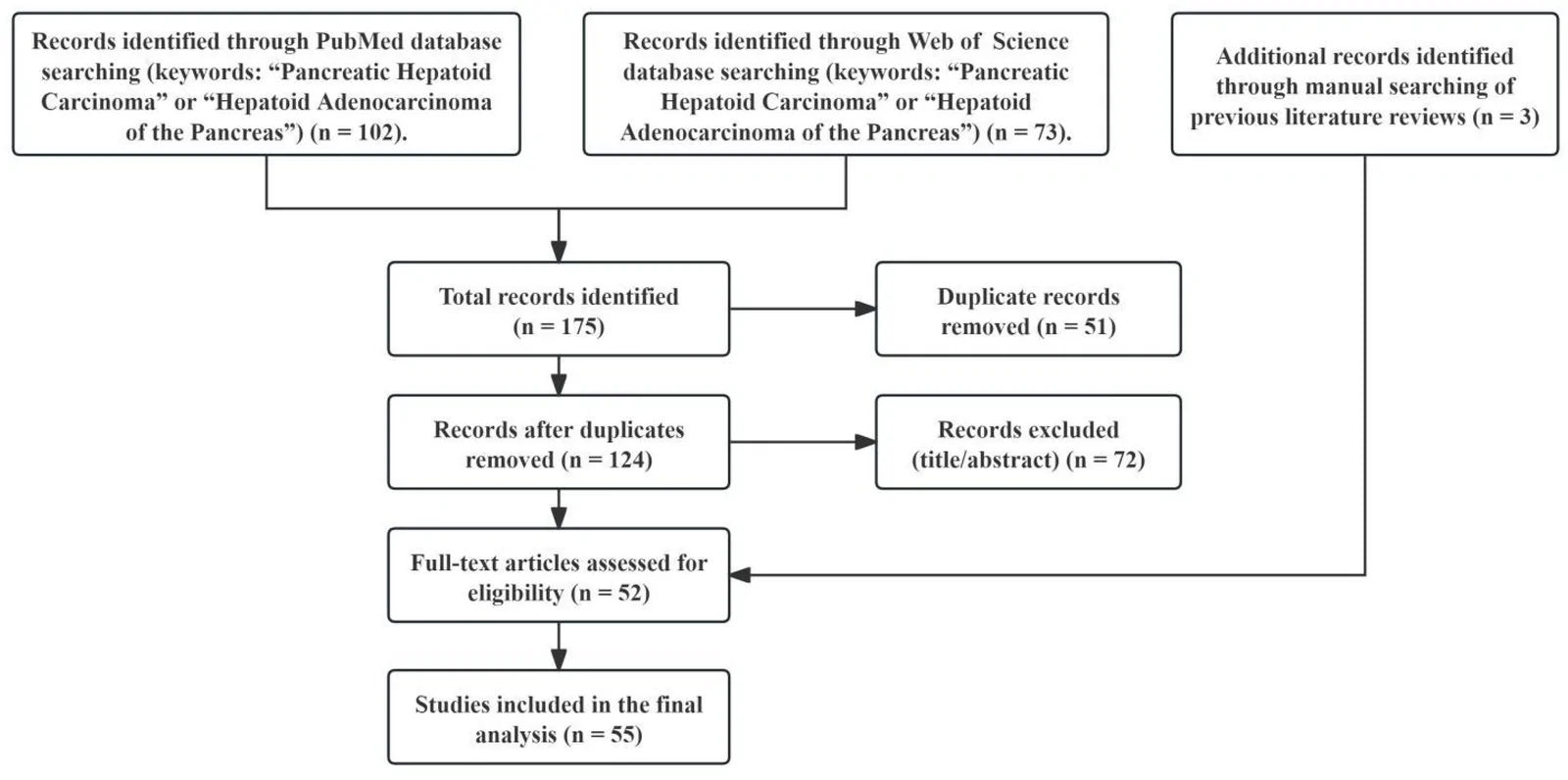
Literature search and study selection process. The initial database search identified 175 records from PubMed and Web of Science. After duplicate removal and title/abstract screening, 52 full-text articles were assessed for eligibility. Three additional studies were identified through manual reference screening. Finally, 55 studies including 57 cases of pancreatic hepatoid carcinoma were included in the systematic analysis.

The initial search identified 175 records. After removal of 51 duplicates, 124 records remained for title and abstract screening. After preliminary screening, 72 ineligible records were excluded, leaving 52 full-text articles for further assessment. In addition, three eligible articles were identified through manual screening of previous reviews and reference lists. Ultimately, 55 studies comprising 57 cases of pancreatic hepatoid carcinoma were included in this systematic analysis. The literature selection process is shown in **Figure 4**.

### Case inclusion and data extraction

To ensure comprehensive case characterization, patient-level data were extracted for each included case, including baseline characteristics, tumor features, serum tumor markers, treatment modalities, and follow-up information (**Table 1**). All data were independently extracted and cross-checked by two investigators. Any discrepancies were resolved by consultation with a third investigator.

**Table 1:** Summary of the clinicopathological characteristics of 57 reported cases of pancreatic hepatoid carcinoma.

CaseNo.	Firstauthor/year (ref.)	Age (years)	Sex	Tumorlocation	Clinicalpresentation	Relevantmedicalhistory	Tumorsize (cm)	TNMstage	Metastatic/involvedsites	Concurrentcomponent	AFPlevel	CA19-9level	CEAlevel	Treatment	Follow-up (months)	Outcome
1	Hruban et al, 1987 (7)	53	F	Tail	Subcutaneous fat necrosis and polyarthritis	Subcutaneous fat necrosis, polyarthritis, and hyperlipidemia	1	pT1N1M1	Liver, lymph node	ACD	Normal	NR	NR	Chemotherapy (5-FU, Adriamycin)	2.75	Died
2	Yano et al, 1999 (8)	57	M	Head	Jaundice, epigastric pain, vomiting and fever	NR	9×7×5	pT3N1M1	Liver, Peritoneum	TGD	Elevated	NR	Elevated	Surgery	3	Died
3	Tanno et al, 1999 (9)	65	F	Body-tail	Epigastric and back pain, anorexia and weight loss	NR	6×5	pT3NxM0	Liver, lymph node	TGD	Elevated	NR	Elevated	Palliative care	6	Died
4	Paner et al, 2000 (10)	28	M	Multifocal	Abdominal and back pain	NR	8×8×6	pT4NxMx	Stomach, ileum and colon	TGD	NR	NR	NR	Surgery, chemotherapy	14	Died
5	Paner et al, 2000 (10)	57	M	Tail	Vomiting, diarrhea, weight loss, diffuse skin rashes and diabetes mellitus	Diabetes mellitus with ischemic optic neuritis	6×4×3.5	pT3NxM1	Liver, lymph node	NED	NR	NR	NR	Surgery, chemotherapy	102	Died
6	Lam et al, 2001 (11)	64	F	Tail	Hypoglycemia, recurrent nocturnal sweating	NR	7×4×4	pT3N1M1	Liver, lymph node	NED	Elevated	NR	NR	Surgery, regional embolization, systemic chemotherapy	22	Died
7	Cuillière et al, 2002 (12)	70	M	Body	Asymptomatic	Adenocarcinoma of prostate	3	NR	No	PHM	Normal	NR	Normal	Surgery	12	Alive
8	Hughes et al, 2004 (13)	51	M	Tail	Asymptomatic	NR	6×5.5×5.5	NR	Liver, lymph node	PHM	Normal	NR	NR	Surgery	14	Alive
9	Matsueda et al, 2006 (14)	49	F	Widespread	Weight loss	Appendicitis	NR	pT3NxM0	Liver	PHM	Elevated	NR	Normal	Surgery, chemotherapy (gemcitabine)	48	Alive
10	Shih et al, 2006 (15)	32	M	Tail	Asymptomatic	Intermittent epigastric pain×10 years	7	pT3N0M0	No	NED	Normal	NR	Normal	Surgery	18	Alive
11	Oh et al, 2006 (16)	21	M	Head	Asymptomatic	No	3.3×2.5×2.5	NR	No	NED	Elevated	NR	NR	Surgery	7	Alive
12	Hameed et al, 2007 (17)	41	F	Head	Gastroesophageal reflux, jaundice, abdominal pain	NR	4.5×4×3	IV, pT3N1M1	Liver, lymph node	NET	Elevated	NR	NR	Surgery, chemotherapy (cisplatin and irinotecan)	27	Died
13	Liu et al, 2007 (18)	80	M	Head	Nausea, diarrhea, weight loss and epigastric palpable mass	NR	5×5×6	pT4NxM0	Transverse colon	PHM	Normal	NR	Normal	Surgery	8	Alive
14	Zhang et al, 2007 (19)	37	F	Widespread	Upper abdominal pain, anorexia, and emaciation	NR	9	pT3NxM1	Duodenum, Liver	NED	Elevated	NR	Elevated	Surgery	3	Died
15	Cardona D et al, 2007 (20)	58	M	Body	Pain in the back and lateral abdomen	Type 2 diabetes and osteoarthritis	3.3×2.5×2.5	pT2N0M0	No	PHM	Normal	NR	NR	Surgery	15	Alive
16	Kubota K et al, 2007 (21)	56	M	Tail	NR	diabetes	6.3×6.2	pT3NxM0	No	PHM	NR	Normal	NR	Surgery	36	Alive
17	Jung et al, 2010 (22)	46	M	Head	Dyspepsia, epigastric palpable mass	No	8×9	IIA, pT3N0M0	Stomach	NED	Elevated	NR	Normal	Surgery	4	Alive
18	Petrelli et al, 2012 (23)	37	F	Body	Epigastric abdominal mass	NR	11	IV, pT3N1M1	Liver, lymph node and lung	TGD	NR	NR	Normal	Targeted therapy (sorafenib)	12	Died
19	Kelly et al, 2012 (24)	53	F	Body-tail	Epigastric pain	NR	NR	pT3N0M0	Liver	PHM	Elevated	NR	NR	Surgery, Chemotherapy (carboplatin and gemcitabine)	22	Alive
20	Kai et al, 2012 (25)	79	F	Tail	Asymptomatic	NR	7×5	III (T3N1M0)	Liver, Left adrenal and Stomach	PHM	NR	NR	Normal	Surgery	2	Died
21	Huang et al, 2012 (26)	52	M	Head	Jaundice, anorexia, and epigastric pain	Type 2 diabetes mellitus	0.5 nodule	pT1N0M0	No	NED	NR	NR	Elevated	Surgery, chemotherapy (sunitinib)	16	Alive
22	Majumder et al, 2013 (27)	60	M	Head	Left upper quadrant pain, jaundice and nausea	NR	5.8×6.0	pT3N1M1	Liver, lymph node	PHM	Normal	NR	NR	Biliary drainage, chemotherapy (GEM)	3	Died
23	Steen S et al, 2013 (28)	61	F	Tail	Asymptomatic	Hypothyroidism, hypertension and gastroesophageal reflux disease	5.3×3.5	pT3N0M0	No	PHM	Normal	Normal	NR	Surgery	60	Alive
24	Xin et al, 2014 (29)	33	F	Head	Asymptomatic	NR	2×1.4×1.8	pT1N0M0	No	NED	Elevated	NR	Normal	Surgery, chemotherapy (GEM)	46	Alive
25	Vanoli et al, 2015 (30)	57	F	Head	Jaundice	hypertension	3.5×3×3	pT3N1M0	Lymph node	PHM	Elevated	NR	Elevated	Surgery, chemotherapy (GEM)	10	Alive
26	Soofi et al, 2015 (31)	69	M	Body-tail	Atypical chest pain	Prostate cancer	5.9	pT3N1M0	No	PHM	Elevated	NR	Normal	Surgery	4	Alive
27	Antonini et al, 2015 (32)	59	M	Body	Weight loss, abdominal discomfort	diabetes	6×5	pT3NxM1	No	PHM	Normal	NR	Normal	Targeted therapy (sorafenib)	4	Died
28	Kuo et al, 2015 (33)	67	M	Tail	Asymptomatic	hypertension	2×2	NR	No	PHM	Normal	NR	Normal	Surgery	6	Alive
29	Veerankutty et al, 2015 (34)	47	M	Tail	Asymptomatic	Nephrolithiasis	3.1×2.9×2.6	pT2N0M0	No	PHM	NR	NR	Normal	Surgery	8	Alive
30	Williams et al, 2015 (35)	71	M	Head	Pancreatitis melena (oozing ulcer at the ampulla)	NR	5	NR	Duodenum, lymph node	PHM	Normal	NR	Normal	Surgery	NR	NR
31	Zhu et al, 2015 (36)	56	M	Body-tail	Asymptomatic	Liver abscess, cholecystolithiasis	5.2×4.8×4.1	T3N0M0	Lymph nodes, liver	NED	Elevated	Normal	Normal	Surgery, regional embolization, Targeted therapy (Sandostatin LAR)	21	Died
32	Stamatova et al, 2016 (37)	78	M	Head	Asymptomatic	NR	8×6	pT3N0M0	No	PHM	Normal	NR	Normal	Surgery	2	Died
33	Chang et al, 2016 (38)	61	M	Body-tail	Asymptomatic	HL, anemia, T2DM, HTN, HLPD, hypoTH, gastric carcinoid	1.3	pT1N0M0	No	PHM	NR	NR	NR	Surgery	6	Alive
34	Akimoto et al, 2016 (39)	59	M	Body	Asymptomatic	NR	5.0×3.5	pT1N0M0	No	PHM	Normal	NR	Normal	Surgery	12	Alive
35	Dogeas E (40)	65	M	Head	Asymptomatic, incidental pancreatic mass	Hypertension; chronic back pain	10	NR	Peripancreatic lymph node	PHM	NR	NR	NR	Surgery, chemotherapy (FOLFIRINOX)	NR	NR
36	Pellini Ferreira et al, 2017 (41)	43	M	Tail	Jaundice, epigastric pain, and watery diarrhea	Hypertension and diabetes	9	pT4NxM1	Liver, Spleen	NED	Normal	NR	Normal	Chemotherapy (capecitabine and temozolomide)	16	Alive
37	Ma et al, 2017 (42)	75	M	Tail	Weight loss	NR	7.8	ypT4N0M0	Liver	PHM	Elevated	NR	Elevated	Neoadjuvant chemotherapy, Surgery	10	Alive
38	Yang et al, 2018 (43)	83	M	Body	Abdominal pain	Prostate cancer	2.7×2.5×1.5	pT2N0M0	No	PHM	NR	NR	NR	Surgery	105	Alive
39	Yang et al, 2018 (43)	72	M	Tail	Severe back pain	NR	12×10.5×4.5	pT3N0M0	No	PHM	NR	NR	NR	Surgery	1	Died
40	Yang et al, 2018 (43)	54	M	Body-tail	Asymptomatic	Rectal cancer	10×9×9	pT4N0M0	Adjacent to transverse colon	PHM	Elevated	NR	Normal	Surgery	29	Died
41	Tomino et al, 2019 (44)	56	M	Head	Asymptomatic	NR	0.7	pT1N0M0	No	PHM	NR	NR	Normal	Surgery	6	Alive
42	Zeng et al, 2020 (45)	36	M	Widespread	Painless jaundice and weight loss	Autoimmune pancreatitis	6.0×7.0	NR	Celiac trunk, Root of transverse mesocolon, Proximal jejunum	PHM	Elevated	NR	Normal	Palliative care	4	Died
43	Chaudhari et al, 2020 (46)	28	M	Head	vomiting, anorexia, abdominal pain	NR	NR	T4bN3bM0	Lymph node	PHM	Elevated	NR	NR	Chemotherapy (5-FU), Targeted therapy (trastuzumab)	16	Died
44	He et al, 2021 (47)	44	F	Body-tail	Upper abdominal pain, vomiting	NR	7.6×7.3	NR	No	PHM	Elevated	NR	Normal	Surgery, TACE, Immunotherapy (Carrilizumab)	8	Alive
45	Trinh et al, 2021 (48)	49	M	Head	Abdominal pain	NR	2.6×2.8	pTxN2M0	Lymph node	NED	Elevated	NR	Normal	Surgery, chemotherapy	12	Alive
46	Wei et al, 2023 (49)	48	M	Head	Asymptomatic	NR	7.5×7×3.7	pT4N1M0	Lymph node	PHM	Elevated	NR	NR	Surgery, Targeted therapy (Sorafenib)	8	Alive
47	Iliesiu A, et al, 2023 (1)	72	F	Presumed pancreatic primary	Severe abdominal pain	Hypertension and Type 2 diabetes	NR	peritoneum	Liver, ovary	PHM	Normal	Elevated	Elevated	Surgery	<1	Died
48	Huang X, 2024 (3)	45	F	Head	Upper abdominal pain, jaundice	NR	4.7×3.5	pT3N?M0	No	PHM	Elevated	NR	Elevated	Surgery, chemotherapy	12	Alive
49	Ahmed Y, 2024 (50)	67	M	Head	poor appetite and weight loss, jaundice	Hepatitis C, Type 2 diabetes mellitus, hypertension, hypothyroidism; hyperlipidemia; GERD.	11.7×7.7×6.1	pT3N2M1	Left supraclavicular lymph node	PHM	Elevated	Normal	NR	Surgery, Targeted therapy (atezolizumab, durvalumab)	16.75	Died (COVID-19)
50	Mi S, 2024 (51)	70	M	Body-tail	Asymptomatic	NR	3×2×2	pT2N0M0	No	PHM	Elevated	NR	NR	Surgery, chemotherapy	1.93	Alive
51	Widdowson KR, 2025 (52)	40	M	Body-tail	NR	NR	1.8	pT3N0M0	No	PHM	NR	NR	NR	NR	NR	NR
52	Dubey A, 2025 (53)	33	F	Body	Upper abdominal pain, palpable epigastric mass	No	8.2×12.2×8.4	pT3/T4N2M0	Lymph node	PHM	Normal	NR	Elevated	chemotherapy	4	Died
53	Wang F, 2025 (2)	43	M	Head	Painless jaundice	Chronic hepatitis B	2.3×2.0	ypT4N0M0	No	PHM	Elevated	Normal	Normal	Surgery, chemotherapy, Targeted therapy (Sintilimab)	NR	Alive
54	Yuan XY, 2025 (54)	45	F	Head	Abdominal pain and jaundice	Hepatitis B, jaundice	3.4×2.9	pT3N2M0	Liver, lymph node	PHM	Elevated	Normal	Normal	Surgery, chemotherapy, Targeted therapy (Sintilimab)	16	Alive
55	Lee D, 2025 (55)	15	F	Whole pancreas	Upper abdominal pain	impaired glucose tolerance	6.4 (head mass)	pT3NxM1	Liver, lymph node	NED	Elevated	Elevated	Normal	Surgery, chemotherapy	10	Died
56	Yang Z, 2025 (56)	46	M	Head	jaundice, poor appetite and weight loss	Schizophrenia, lung cancer	4.0×2.5	pT2N1Mx	Lymph node	NED	Elevated	Elevated	NR	Surgery	4	Alive
57	Punnen S, 2025 (57)	42	F	Body	Upper abdominal pain	No	8.6×7.9×6.5	pT3N0M0	No	PHM	Elevated	Normal	Normal	Surgery, chemotherapy	9	Alive

## Clinicopathological characteristics of the included PHC cases

A total of 57 previously reported cases of pancreatic hepatoid carcinoma (PHC) were included in this systematic analysis. The median age of the patients was 54 years, with an age range of 15–83 years, and most patients were male (38/57, 66.7%). Regarding clinical presentation, the most common symptom was abdominal or back pain (22/57, 38.6%), followed by asymptomatic presentation (18/57, 31.6%), weight loss/anorexia/poor appetite (12/57, 21.1%), and jaundice (12/57, 21.1%).

The tumor was most commonly located in the pancreatic body and tail (30/57, 52.6%), followed by the pancreatic head (21/57, 36.8%). Six cases presented with multifocal, diffuse, or whole-pancreas involvement (10.5%). Elevated serum AFP levels were reported in 28 patients (49.1%). Histologically, 38 cases (66.7%) were classified as pure hepatoid carcinoma or had no reported mixed components. Neuroendocrine, ductal/adenocarcinoma, and acinar cell components were reported in 14, 4, and 1 cases, respectively.

Metastatic or involved sites were reported in 33 patients (57.9%), with lymph nodes and the liver being the most frequently affected sites. Regarding treatment, most patients underwent surgery alone or surgery combined with other treatment modalities. At the last follow-up, 32 patients were alive, 21 had died, 1 had died from a non-tumor-related cause, and outcome data were unavailable for 3 patients (**Table 2**).

**Table 2:** Clinical and biochemical characteristics of 57 cases of pancreatic hepatoid carcinoma.

Variable	Number of cases (%)
Age (years)	
Median (range)	54 (15–83)
≥50	33 (57.89)
	24 (42.11)
Sex	
Male	38 (66.67)
Female	19 (33.33)
Clinical presentation	
Asymptomatic	18 (31.58)
Abdominal/back pain	22 (38.60)
Weight loss/anorexia/poor appetite	12 (21.05)
Jaundice	12 (21.05)
Abdominal/epigastric mass	5 (8.77)
Gastrointestinal symptoms	10 (17.54)
Hypoglycemia	1 (1.75)
Skin/fat necrosis or polyarthritis	2 (3.51)
Other/unclear	3 (5.26)
Location	
Head	21 (36.84)
Body or tail	30 (52.63)
Multifocal/widespread/whole pancreas	6 (10.53)
Serum AFP level	
Elevated	28 (49.12)
Normal	16 (28.07)
NR	13 (22.81)
Serum CA19–9 level	
Elevated	3 (5.26)
Normal	7 (12.28)
NR	47 (82.46)
Serum CEA level	
Elevated	9 (15.79)
Normal	26 (45.61)
NR	22 (38.60)
Concurrent component	
Pure hepatoid carcinoma/no concurrent component	38 (66.67)
Neuroendocrine component	14 (24.56)
Ductal/glandular adenocarcinoma component	4 (7.02)
Acinar cell component	1 (1.75)
Metastatic/involved sites	
No metastatic/involved site reported	24 (42.11)
Any metastatic/involved site reported	33 (57.89)
Liver	19 (33.33)
Lymph node	20 (35.09)
Peritoneum	1 (1.75)
Lung	1 (1.75)
Other sites	10 (17.54)
Treatment	
Surgery only	25 (43.86)
Surgery combined with other therapy	22 (38.60)
Non-surgical systemic/local therapy	7 (12.28)
Palliative/supportive care only	2 (3.51)
NR/unclear	1 (1.75)
Outcome at last follow-up	
Alive	32 (56.14)
Died	21 (36.84)
Died of non-cancer cause	1 (1.75)
NR	3 (5.26)

**Figure 5:**
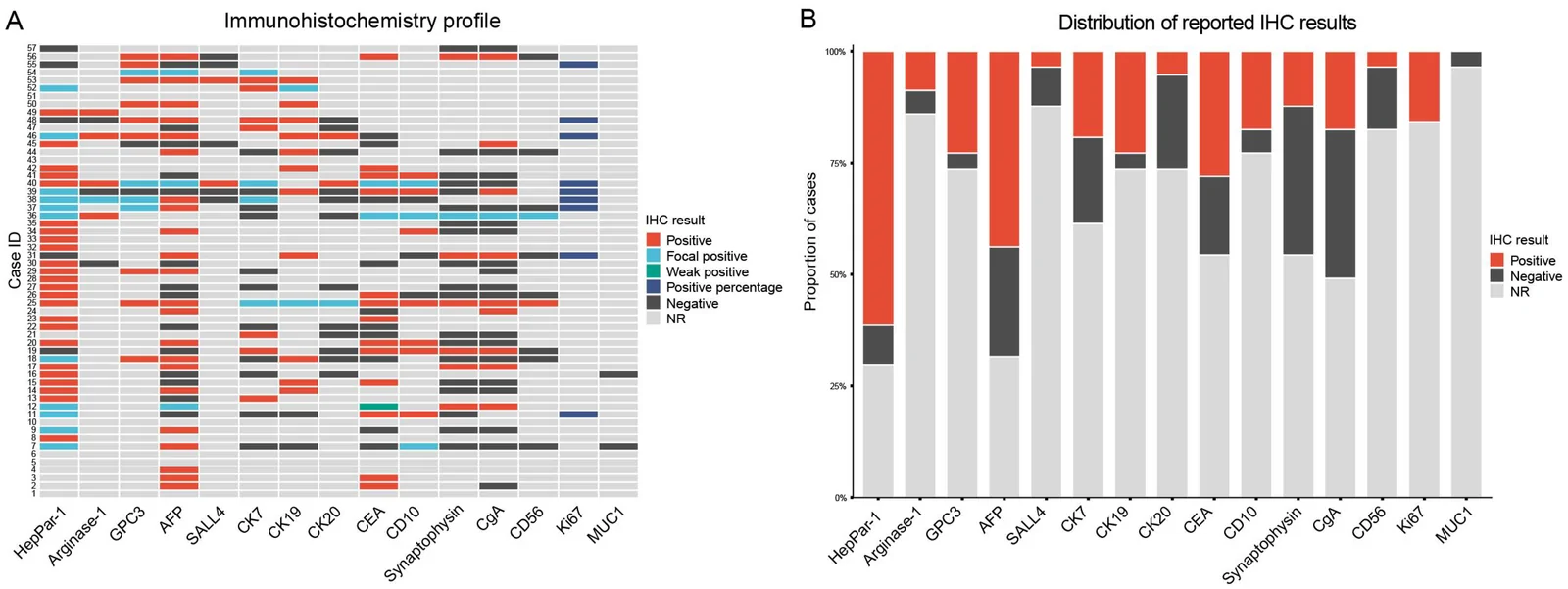
Immunohistochemical heterogeneity of pancreatic hepatoid carcinoma. **(A)** Heatmap showing the immunohistochemical staining results of representative markers reported in 57 pancreatic hepatoid carcinoma cases. Each row represents one case, and each column represents one marker. **(B)** Stacked bar plot showing the distribution of positive, negative, and not-reported results for each immunohistochemical marker among all 57 included cases. For each marker, the positive, negative, and not-reported proportions were calculated using the total number of included cases as the denominator. Focal, weak, and partial positivity were grouped into the positive category in the stacked bar plot. “Not reported” included cases in which the marker was not tested, not mentioned, or for which no interpretable result was available. These proportions represent case-level reporting frequencies in the published literature and do not indicate the percentage of positively stained tumor cells within individual specimens. IHC, immunohistochemistry; NR, not reported or unavailable.

Based on the clinicopathological characteristics described above, we further summarized the immunohistochemical findings of the 57 PHC cases. As shown in **Figure 5A**, the markers examined in previous reports mainly included HepPar-1, Arginase-1, GPC3, AFP, SALL4, CK7, CK19, CK20, CEA, CD10, synaptophysin, CgA, CD56, Ki-67, and MUC1. **Figure 5B** presents the case-level distribution of positive, negative, and not-reported results for each marker among all 57 included cases. Markers associated with hepatoid differentiation, such as HepPar-1, Arginase-1, GPC3, and AFP, were positive in some cases, supporting hepatocellular-like differentiation; however, positive expression of these markers was not observed in all cases. Meanwhile, some tumors expressed pancreatobiliary epithelial markers, including CK7, CK19, CEA, and MUC1, as well as neuroendocrine-related markers such as synaptophysin, CgA, and CD56, suggesting a certain degree of immunophenotypic heterogeneity in PHC. A high Ki-67 index was reported in some cases, indicating that PHC may exhibit substantial proliferative activity.

However, because the immunohistochemical panels and interpretation criteria varied across studies, and because a considerable proportion of markers were not reported in many cases, the positive rate of any single marker should not be overinterpreted. Overall, the diagnosis of PHC should be based on an integrated assessment of hepatocellular-like morphology, expression of hepatoid differentiation markers, evidence supporting a primary pancreatic origin, serological features, and exclusion of primary hepatocellular carcinoma.

## Metastatic patterns and treatment modalities

Among the 57 patients, 24 cases had no clearly reported metastatic or involved sites, accounting for 42.11%, whereas 33 cases had metastatic or involved sites, accounting for 57.89%. Lymph node involvement was reported in 20 cases (35.09%), and liver involvement was reported in 19 cases (33.33%). In a small number of cases, involvement of the peritoneum, lung, or other sites was also observed. These findings suggest that lymph node metastasis and liver involvement are not uncommon at the time of PHC diagnosis. Therefore, clinical evaluation should not be limited to the primary pancreatic lesion, but should also include comprehensive staging of lymph nodes, the liver, and other potential distant metastatic sites.

**Figure 6:**
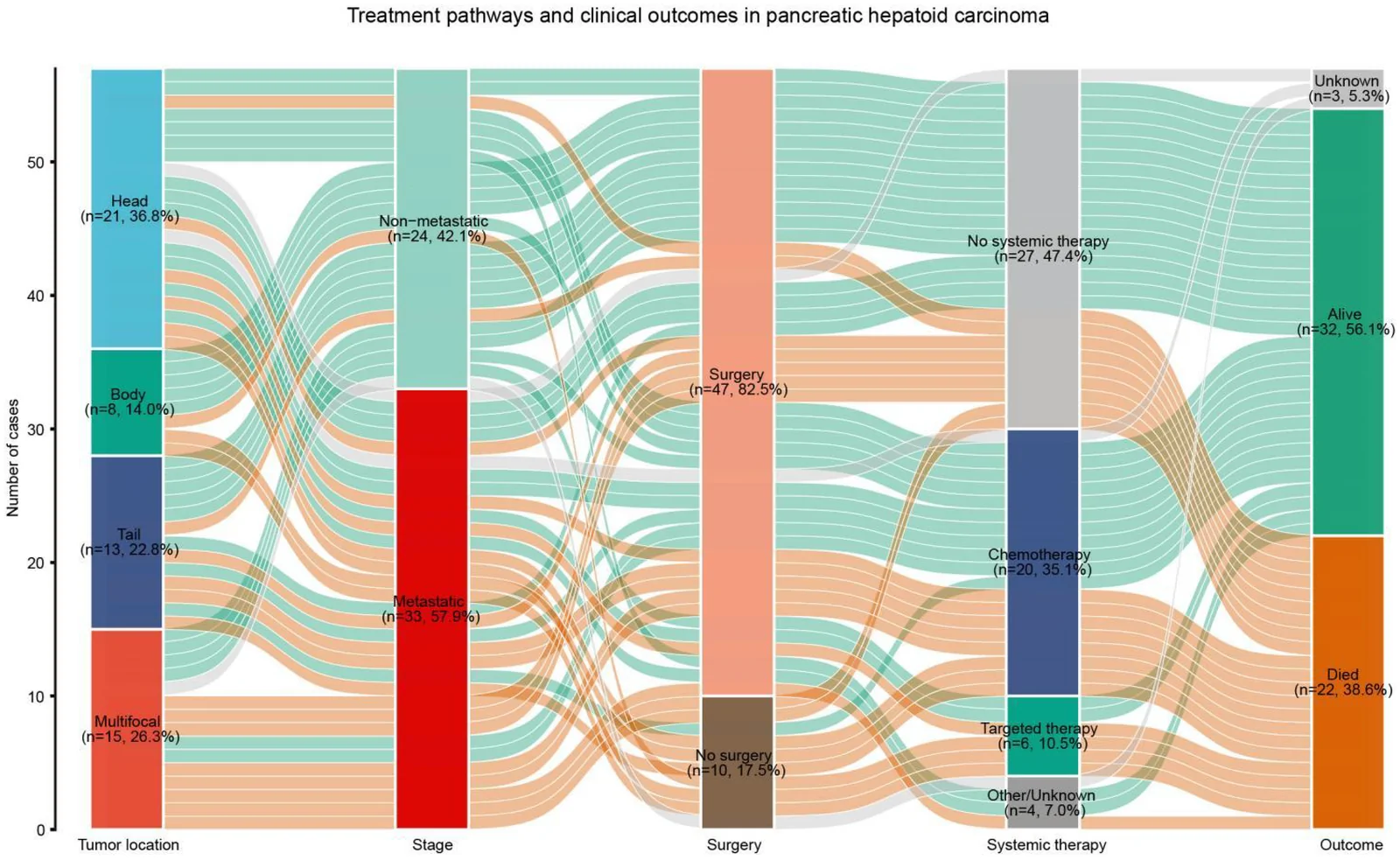
Sankey diagram of treatment pathways and outcomes in pancreatic hepatoid carcinoma. The diagram summarizes 57 reported cases according to tumor location, metastatic status, surgical treatment, systemic therapy, and final outcome. Flow widths represent the number of cases in each category. The analysis shows that metastatic disease was common at diagnosis, surgery remained the predominant treatment approach, and systemic therapy was reported in a limited proportion of cases. PHC, pancreatic hepatoid carcinoma.

Regarding treatment modalities, 25 patients underwent surgery alone, accounting for 43.86%; 22 patients received surgery combined with other treatments, accounting for 38.60%; 7 patients received nonsurgical systemic or local treatment, accounting for 12.28%; and 2 patients received only palliative or supportive care, accounting for 3.51%. The treatment modality was unclear in 1 case. Further visualization of the treatment pathways using a Sankey diagram (**Figure 6**) showed that surgery remained the most frequently reported treatment approach, particularly among patients with resectable or localized disease.

However, because more than half of the patients had metastatic or involved sites, surgery alone may not adequately address all clinical scenarios. With regard to systemic therapy, some patients received chemotherapy or targeted therapy, but the overall proportion remained limited, suggesting that evidence supporting systemic treatment for PHC is still insufficient. In terms of outcomes, at the last follow-up, 32 patients were alive, 21 had died of tumor-related causes, 1 had died of a non-tumor-related cause, and outcome data were unavailable for 3 patients.

## Survival outcomes and prognostic analysis

**Figure 7:**
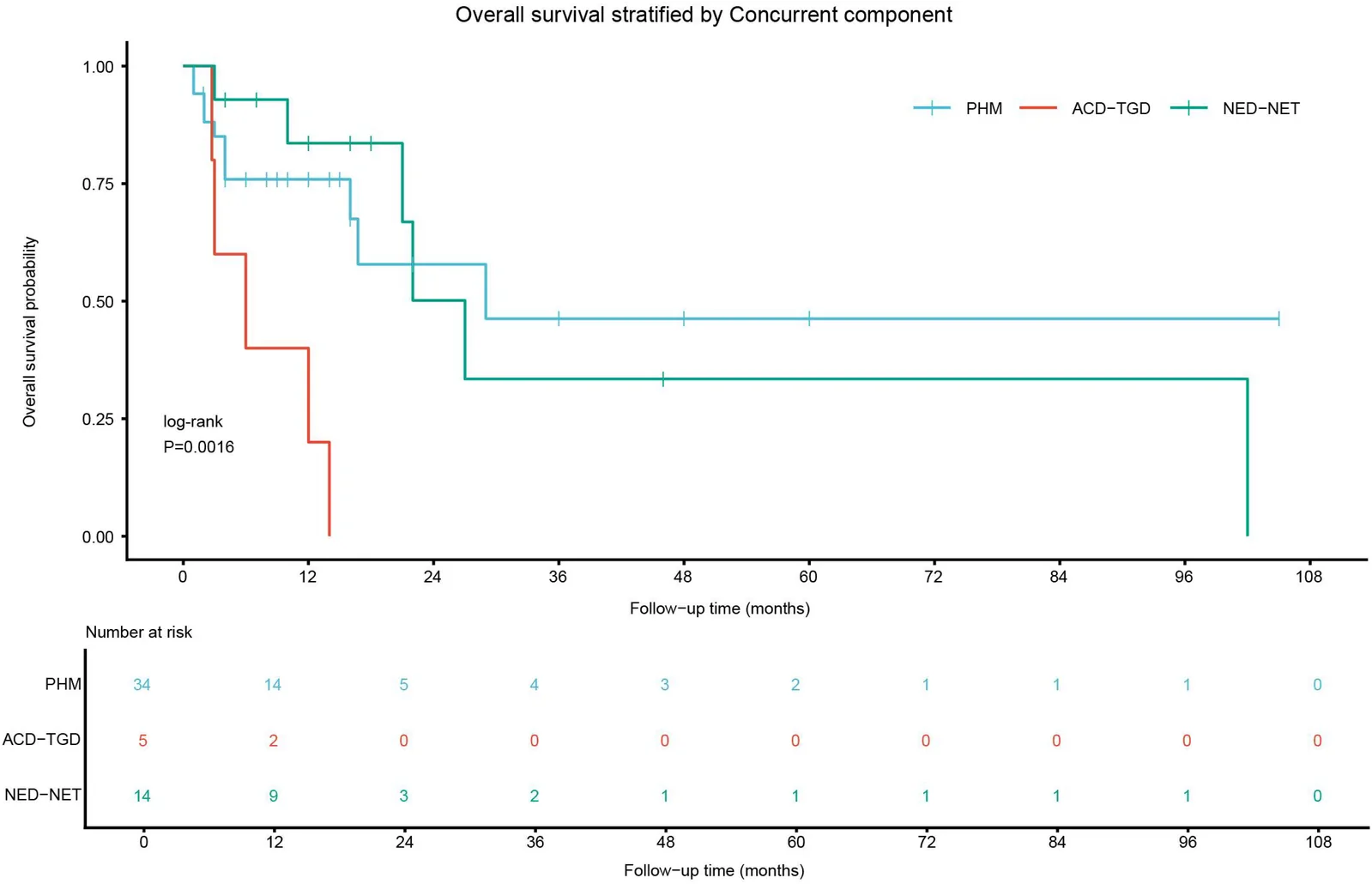
Kaplan–Meier analysis of overall survival according to concurrent histological components. Overall survival curves of pancreatic hepatoid carcinoma cases stratified by reported pathological composition. Tick marks indicate censored cases, and the number-at-risk table is displayed below the plot. The curves were generated from survival information available in published case reports and small case series. Because of the small subgroup sizes, heterogeneous diagnostic criteria and treatments, variable follow-up duration, and incomplete outcome reporting, these estimates are descriptive and hypothesis-generating and should not be interpreted as demonstrating definitive prognostic differences or population-level survival probabilities. PHM, pure hepatoid morphology; ACD–TGD, acinar cell differentiation/tubular or glandular differentiation; NED–NET, neuroendocrine differentiation/neuroendocrine tumor.

After stratification by accompanying histological components (**Figure 7**), the survival curves showed certain differences among different pathological subtypes. The overall log-rank test indicated a statistically significant difference in survival distributions among the three groups (P = 0.0016).Compared with cases of pure hepatoid carcinoma or those without clearly reported accompanying components, cases with ductal/adenocarcinoma-like or acinar cell components appeared to show an earlier decline in the Kaplan–Meier curves. Cases with neuroendocrine components appeared to exhibit a relatively distinct survival pattern. However, given the small number of cases in some subgroups and the heterogeneity of treatment modalities, follow-up duration, and outcome reporting across previous studies, the statistically significant global log-rank result should be interpreted cautiously, and these findings should be regarded as descriptive and hypothesis-generating rather than evidence of definitive or independent prognostic differences among histological subtypes. Moreover, these Kaplan–Meier estimates were derived from available published cases and should not be interpreted as population-level survival probabilities for PHC.

In combination with the treatment pathway analysis described above, metastatic or involved sites were reported in a substantial proportion of PHC cases, whereas surgery remained the most frequently adopted treatment modality in the literature. It should be noted that patients who underwent surgery were generally more likely to have resectable disease or a lower tumor burden. Therefore, the relatively favorable survival observed in surgically treated cases may have been influenced by selection bias and confounding by indication and cannot be simply interpreted as evidence that surgery itself confers a causal survival benefit or is applicable to all patients with PHC. For patients with resectable disease, radical resection remains an important therapeutic option. For those with locally advanced, metastatic, or recurrent disease, systemic treatment should be considered early based on multidisciplinary evaluation, and molecular profiling should be incorporated to explore potential individualized therapeutic opportunities.

## Summary of the systematic analysis

This systematic analysis showed that PHC lacks specific clinical manifestations, exhibits heterogeneous pathological compositions, and is frequently associated with metastasis or local involvement at initial diagnosis. Elevated serum AFP may provide an important diagnostic clue, but it is not observed in all cases. Therefore, diagnosis should still rely on an integrated assessment of histological morphology, immunophenotypic features, and radiological findings.

In terms of treatment, surgery remains the main therapeutic approach for patients with resectable disease. However, systemic treatment strategies for patients with locally advanced or metastatic PHC have not yet been standardized. Considering the clinical benefit achieved with targeted therapy in the present case, early molecular profiling may help identify potentially actionable targets and provide a basis for individualized treatment of PHC.

## Discussion

The diagnostic process in the present case is representative to some extent. The patient initially presented with obstructive jaundice, and imaging revealed a pancreatic head-neck mass accompanied by portal vein tumor thrombus and regional lymph node metastases, indicating aggressive disease at diagnosis (3, 19). Serologically, AFP was markedly elevated, whereas CA19–9 was only mildly increased, a pattern consistent with the clinical features of hepatoid tumors (9, 14). Pathologically, biopsy revealed poorly differentiated carcinoma with hepatocellular-like morphology. Immunohistochemistry showed positivity for AFP, SALL4, CK, CK19, and synaptophysin, with negativity for CK7, CD56, CEA, and INSM1, and the Ki-67 proliferation index was approximately 75%. The intact expression of mismatch repair proteins indicated pMMR/MSS status, and PD-L1 expression was low. In the absence of radiological evidence supporting primary hepatocellular carcinoma in the liver, and given the lack of sufficient support for typical pancreatic ductal adenocarcinoma or pancreatic neuroendocrine tumor, the final diagnosis of PHC was considered reasonable. Importantly, the diagnosis of PHC should not be excluded solely because of an incomplete hepatocellular marker profile, as both previous reports and the IHC summary in the present study suggest that hepatocellular differentiation-associated markers show an inconsistent expression pattern in PHC (29, 35).

With regard to treatment, there is currently no established standard regimen for PHC. Previously reported cases have often been treated by referring to therapeutic strategies for conventional pancreatic cancer, hepatocellular carcinoma, or gastrointestinal hepatoid adenocarcinoma; however, treatment efficacy has varied substantially, and prospective evidence remains lacking. In the present study, treatment pathway analysis showed that 47 of the 57 patients underwent surgery, suggesting that surgery remains the main treatment modality for resectable PHC (13, 17). Nevertheless, 33 patients had metastatic disease at initial diagnosis, indicating that a considerable proportion of patients were not suitable candidates for upfront curative surgery at the time of diagnosis. Further survival analysis showed that distant metastasis at diagnosis and absence of surgical treatment were associated with poorer overall survival. These findings are consistent with the aggressive nature and early dissemination tendency of PHC. However, the association between surgery and survival should be interpreted with caution because of potential selection bias: patients eligible for surgery are generally more likely to have a lower tumor burden, more favorable disease stage, and better performance status. Therefore, surgery should be considered an important therapeutic opportunity for patients with resectable disease, rather than a universally applicable strategy for all patients with PHC.

The clinical course of the present patient further illustrates the therapeutic complexity of advanced PHC. At diagnosis, the patient had stage IV disease with portal vein tumor thrombus and multiple metastatic lymph nodes, making upfront surgical resection unsuitable. After biliary drainage improved obstructive jaundice and liver function, he received an exploratory first-line regimen consisting of pembrolizumab, lenvatinib, paclitaxel, and S-1. However, after two cycles, serum AFP increased from 12,457 ng/mL to 18,000 ng/mL, and imaging showed rapid enlargement of the pancreatic primary lesion and metastatic lymph nodes, with some lymph nodes increasing by approximately 40%. This pattern was clinically considered consistent with immune checkpoint inhibitor-related hyperprogressive disease. Although causality cannot be confirmed from a single case, the coexistence of pMMR/MSS status, low tumor mutational burden, low PD-L1 expression, and MDM2 amplification may partly explain the limited benefit from immunotherapy and the rapid disease acceleration observed in this patient.

The most clinically significant aspect of this case is the identification of a BRAF V600E mutation and the deep response achieved with dual BRAF/MEK inhibition after failure of first-line therapy. BRAF is a key kinase in the RAS–RAF–MEK–ERK/MAPK signaling pathway. The BRAF V600E mutation leads to constitutive activation of MAPK signaling, thereby promoting tumor cell proliferation and survival. In multiple non-melanoma solid tumors, BRAF V600E has been established as an actionable therapeutic target. For example, Hyman et al. demonstrated in a multicancer study that patients with BRAF V600E-mutant solid tumors could benefit from BRAF inhibitor therapy (58). Furthermore, the ROAR basket study showed that dabrafenib plus trametinib exhibited antitumor activity and manageable safety in multiple rare BRAF V600E-mutant cancers, providing important evidence supporting the tumor-agnostic application of dual BRAF/MEK inhibition (59, 60).

In PHC, evidence regarding BRAF V600E and its value as a therapeutic target remains extremely limited. Chang et al. previously performed next-generation sequencing analysis in PHC, suggesting that molecular profiling may provide potential therapeutic clues for this rare tumor; however, actionable targets and their corresponding treatment efficacy have not been systematically validated (61). In the present case, comprehensive genomic profiling revealed BRAF V600E with a variant allele frequency of 31.89%, together with MDM2 and MYC amplification, low tumor mutational burden, MGMT promoter methylation, and pMMR/MSS status. Previous pan-cancer studies have suggested that MDM2 amplification may be associated with unfavorable outcomes and accelerated disease progression during immune checkpoint inhibitor treatment, potentially through attenuation of p53 function and modulation of the tumor immune microenvironment (62, 63). However, the available evidence is mainly derived from retrospective analyses and limited case series and does not establish a causal relationship between MDM2 amplification and immunotherapy-associated hyperprogression. Therefore, in this patient, MDM2 amplification may be considered a possible contributing factor to the rapid progression observed during the initial immunotherapy-containing regimen, but it cannot be regarded as the sole or definitive explanation. After treatment with dabrafenib plus trametinib, serum AFP decreased rapidly within 1 week and returned to the normal range after approximately 2 months. PET/CT subsequently showed marked shrinkage of the pancreatic lesion to approximately 0.7 cm, with regression or disappearance of the portal vein tumor thrombus and metastatic lymph nodes. Plasma ctDNA-based molecular residual disease assessment performed in August 2024 showed no detectable molecular residual signal, corresponding to an MRD-negative result. Peripheral blood minimal residual disease also became negative, and the best response was assessed as partial response (38). The high consistency among molecular findings, AFP dynamics, radiological response, and the initial conversion of ctDNA-based MRD to an undetectable level supports the possibility that BRAF V600E acted as a clinically relevant driver alteration in this case. However, subsequent ctDNA-based monitoring showed re-emergence and an increase in the molecular residual signal, indicating that MRD negativity was not sustained. These findings should be interpreted as complementary molecular monitoring results rather than independent evidence of radiographic progression or complete tumor eradication.

This case also provides clinically useful information on acquired resistance and subsequent local treatment. After approximately 6 months of BRAF/MEK-targeted therapy, AFP gradually increased again, suggesting occult residual disease or early progression. Subsequent treatment adjustments, including dabrafenib dose escalation, cetuximab addition, and replacement of trametinib with tunlametinib, failed to achieve durable tumor marker control. This clinical course suggests that resistance to MAPK pathway inhibition may emerge even after a deep initial response. Because no standard salvage regimen has been established for progressive PHC, these treatment modifications were individualized after multidisciplinary evaluation. Dabrafenib dose escalation was an empirical attempt to strengthen BRAF pathway suppression, given the previous marked response and acceptable treatment tolerance. Cetuximab was added based on evidence from other BRAF V600E-mutant gastrointestinal tumors showing that compensatory EGFR activation may restore MAPK signaling after BRAF inhibition (64–66). In contrast, the available genomic findings did not identify actionable FGFR alterations or compelling cell-cycle abnormalities to support FGFR or CDK4/6 inhibition. The subsequent replacement of trametinib with tunlametinib was intended to maintain MEK inhibition in the setting of persistent biochemical disease activity. These modifications were exploratory and were not supported by direct molecular confirmation of the underlying resistance mechanism. Based on evidence extrapolated from studies of other BRAF V600E-mutant malignancies, potential resistance mechanisms may include MAPK pathway reactivation driven by enhanced RAS signaling, BRAF amplification or aberrant splicing, RAF dimerization, and secondary alterations in MEK1/2. Activation of bypass signaling pathways involving EGFR, PI3K–AKT–mTOR, FGFR, or SHP2, as well as the selective expansion of BRAF-independent tumor subclones, may also contribute to resistance (65, 67–71). However, because no repeat biopsy or longitudinal molecular profiling was performed at progression, these mechanisms remain biologically plausible hypotheses rather than alterations demonstrated in this patient. Nevertheless, local treatment provided meaningful disease control in this patient. Proton radiotherapy was delivered to the residual pancreatic lesion after FAPI PET/CT suggested persistent local metabolic activity, and AFP returned to the normal range during radiotherapy. Later, CyberKnife stereotactic radiotherapy was used to treat a newly detected 2.3-cm caudate liver metastasis, after which local lesions remained controlled and AFP decreased to 14 ng/mL. These findings suggest that, for selected patients with advanced PHC who achieve systemic disease control but develop oligoresidual or oligoprogressive lesions, local radiotherapy may serve as an important component of multidisciplinary management.

## Data Availability

The original data supporting the findings of this study are included in the article and Supplementary Material. Additional clinically relevant data are available from the corresponding author upon reasonable request.
